# 

**Published:** 1866-06

**Authors:** 


**E. L. HOLMES, M D., H. M. LYMAN, M D., R. M. LACKEY, M.D., Editors.**

All letters for the JOURNAL should be addressed to R. M. LACKEY, M.D., P. O. Box 2175, Chicago, Ill. *Office*—169 *South Dearborn Street.*

**TERMS—$2 PER ANNUM, IN ADVANCE.**

$3 if not paid before 1st of July of each year. Single Copies, 25 cents.

